# Computer‐Aided Diagnosis in the Evaluation of Thyroid Nodules: A Study of Intra‐ And Inter‐Rater Reliability and Agreement

**DOI:** 10.1002/hed.70264

**Published:** 2026-04-06

**Authors:** Leonardo de Souza Piber, Luis Carlos Uta Nakano, Carolina Dutra Queiroz Flumignan, Ronald Luiz Gomes Flumignan

**Affiliations:** ^1^ Evidence‐Based Health Universidade Federal de São Paulo São Paulo Brazil; ^2^ Division of Vascular and Endovascular Surgery, Department of Surgery Universidade Federal de São Paulo São Paulo Brazil; ^3^ Discipline of Vascular and Endovascular Surgery Centro Universitário São Camilo São Paulo Brazil

**Keywords:** computer‐aided diagnosis, inter‐rater agreement, reliability, thyroid nodule, ultrasonography

## Abstract

**Objective:**

To evaluate the intra‐ and inter‐rater reliability of a computer‐aided diagnosis system applied to thyroid nodule assessment.

**Methods:**

This prospective, single‐center study included 150 thyroid nodules evaluated by two physicians at two time points, 90 days apart. Analyses were performed using the AmCAD‐UT system, focusing on morphological features and ACR TI‐RADS classification. Cohen's kappa coefficient and percentage agreement were used to assess reliability.

**Results:**

Intra‐rater reliability ranged from moderate to almost perfect, with kappa values from 0.49 (95% CI: 0.31–0.66) to 0.98 (95% CI: 0.96–1.00), and agreement rates from 81.3% to 99.3%. Rater 2 demonstrated higher reproducibility across most variables, particularly for “texture” (*k* = 0.98), “margin” (*k* = 0.90), “composition” (*k* = 0.93), and “taller‐than‐wide” (*k* = 0.92). Inter‐rater agreement was more variable, with kappa values ranging from 0.43 (95% CI: 0.23–0.62) to 0.96 (95% CI: 0.89–1.00), and agreement percentages from 78.0% to 99.3%. The lowest inter‐rater reproducibility was observed for “shape”.

**Conclusion:**

The computer‐aided diagnosis system demonstrated predominantly moderate to almost perfect intra‐rater reliability and moderate to strong inter‐rater agreement across most evaluated features. The highest reproducibility was observed for “taller‐than‐wide,” “texture,” and “composition,” whereas “shape” consistently showed lower agreement. These findings support the system's role as a reliable adjunct for standardizing thyroid nodule assessment, although its performance remains partially influenced by operational factors and warrants further multicenter validation.

## 1 Introduction

The device evaluated in this study was the AmCAD‐UT Detection v2.2 computer‐aided diagnostic system (AmCad BioMed Corporation, Taiwan). According to the manufacturer's documentation, AmCAD‐UT Detection is a commercially available CAD software registered with the Brazilian National Health Surveillance Agency (ANVISA; registration n^o^. 81309969001) and cleared by the U.S. Food and Drug Administration through the 510(k) pathway (K180006), designed to analyze B‐mode still ultrasound images of thyroid nodules by extracting and objectively quantifying morphological characteristics [1, 2, 3]. The system automatically assigns scores based on the ACR TI‐RADS criteria [4, 5], assisting in the stratification of the malignancy risk of the nodules. One hundred and fifty thyroid nodules were studied, from patients of both sexes, aged over 18, referred for fine‐needle aspiration (FNA) [6] in a private imaging diagnostic service in the city of São Paulo. The analyses were conducted by two physicians specialized in ultrasound, both with over 10 years of experience in thyroid diagnosis and specific training in the use of the AmCAD‐UT system, in addition to specialist titles, ensuring the standardization of the evaluations.

Computer‐aided diagnosis (CAD) systems have been extensively investigated as auxiliary tools in the diagnosis of thyroid nodules, especially for their ability to reduce inter‐rater variability and support less experienced professionals [7]. Recent systematic reviews confirm that these systems exhibit high diagnostic sensitivity and promising performance in risk stratification. The meta‐analysis by Xue et al., which included 25 studies with over 17 000 ultrasound images, demonstrated a pooled sensitivity of 88% (95% CI: 85%–90%), specificity of 81% (95% CI: 74%–86%), and an area under the ROC curve (AUC) of 0.92 (95% CI: 0.89–0.94). These results were particularly striking in patients under 50 years old and indicate that CADs can offer accuracy comparable to that of specialists in many contexts [8]. Complementarily, the meta‐analysis conducted by Zhao et al., based on five clinical studies, reported a sensitivity of 87% (95% CI: 73%–94%), a specificity of 79% (95% CI: 63%–89%), and an area under the ROC curve of 0.90, indicating performance similar to that of experienced radiologists in terms of malignancy detection [9]. However, both studies highlight that the specificity of CADs is still lower than that of specialists, which can result in a higher number of false positives, especially in nodules with complex morphological characteristics. Nevertheless, the authors emphasize that CADs have significant clinical value as a decision support tool, especially in environments with less expertise or for radiologists in training [8, 9]. Multicenter prospective studies with real‐time analysis continue to be recommended to consolidate their use in clinical practice.

Most research on reliability and agreement has focused only on comparing performance between CAD and doctors, without adequately addressing intra‐rater stability over time. Moreover, in Brazil, there is no data on the reliability and concordance of AmCAD‐UT applied to ultrasound images of thyroid nodules. Thus, the present study was developed to fill this gap, investigating for the first time both the intra‐ and inter‐rater reliability and agreement of the system. These data are fundamental to validate the use of AmCAD‐UT as a diagnostic support tool and to establish its role in clinical practice.

The objective was to study the reliability and intra‐ and inter‐rater agreement of thyroid nodule analyses by ultrasound physicians using computer‐aided diagnosis (AmCAD‐UT, AmCad BioMed, Taiwan).

## 2 Methods

This prospective study was designed to assess intra‐ and inter‐rater reliability of CAD‐based thyroid nodule characterization and ACR TI‐RADS classification.

A total of 150 thyroid nodules were included in the study, from patients of both sexes, with a minimum age of 18 years and no maximum age limit, obtained from ultrasound examinations at the time of FNA of the thyroid nodules in a private imaging diagnostic service in São Paulo. Fine‐needle aspiration (FNA) had been requested by the patients' treating physicians as part of routine clinical care prior to study enrollment. The study did not influence the indication for FNA, and CAD analyses were performed for research purposes only. Information on whether the referring clinicians based the FNA request on ACR TI‐RADS criteria was not consistently available. The selection was made through consecutive and convenience sampling, to reflect real clinical practice. There were no losses of cases during the study period. All participants signed the Free and Informed Consent Term. The study was approved by the Research Ethics Committee of the Federal University of São Paulo (CAAE: 35820220.0.0000.5505), with an opinion issued on February 4, 2021, in accordance with the ethical standards in force for research involving human beings.

The ultrasound physician responsible for performing the FNA captured three ultrasound images in B‐mode for each nodule, aiming to encompass the main morphological characteristics required by the ACR TI‐RADS classification [10]. All ultrasound examinations were performed using a GE Logiq 7 system (GE Healthcare, Milwaukee, WI, USA) equipped with a high‐frequency linear transducer operating at 15 MHz. Standard B‐mode settings were used, and image acquisition parameters were adjusted according to routine clinical practice to optimize visualization of thyroid morphology. The operator selected the image that best characterized the nodule according to the ACR TI‐RADS classification, in transverse or longitudinal acquisition, depending on the predominant characteristic of the nodule. The images were saved in JPEG format, without patient identification, and sequentially numbered. Next, the images and the data collection form were sent to the responsible research physician. The responsible researcher individually forwarded the images to two evaluating doctors via email for analysis on the AmCAD‐UT platform.

The CAD system evaluated was AmCAD‐UT Detection v2.2 (AmCad BioMed, Taiwan), a commercially available AI‐based computer‐aided diagnosis platform designed to assist thyroid nodule characterization using B‐mode still ultrasound images. According to the manufacturer's documentation, the system incorporates deep learning technology for automatic nodule detection and performs quantitative analysis of sonographic features following user‐defined region‐of‐interest delineation. It generates structured outputs aligned with the ACR TI‐RADS lexicon, including feature‐level classification and overall risk stratification. Detailed information regarding the internal model architecture and training datasets is proprietary and not publicly disclosed. The present study was designed to evaluate the reproducibility of the system outputs under standardized clinical conditions rather than its intrinsic algorithmic performance. The platform was selected based on its regulatory approval (FDA and ANVISA clearance) and its availability in our clinical setting. The aim of this study was not to compare different CAD platforms, but to evaluate the reliability of AmCAD‐UT within the Brazilian clinical context, where data on its reproducibility are currently lacking. The software supports analysis of DICOM files and common image formats [1, 2]; in this study, images were analyzed in JPEG format due to workstation compatibility. After image upload, manual delineation of the nodule contour initiates automated feature extraction and TI‐RADS–based risk categorization [1]. The evaluating doctors separately accessed the platform and performed the CAD assessment for each nodule, and screenshots of the results were sent to the responsible researcher. Since the measurement of the largest axis of the nodule must be considered for the ACR TI‐RADS recommendation, and this measurement was not evident in the image chosen for analysis in some cases, the researcher provided the raters with the nodule measurements. To avoid memory bias, the data were deleted from the platform the day after the analysis. After 90 days, the same images, with new identification numbers, were resent to the evaluating doctors for a repeat analysis on the AmCAD‐UT platform. Again, the results were sent to the researcher. In case of doubt or disagreement during the analyses, a third evaluating physician was consulted to establish consensus.

Two radiologists dedicated to ultrasonography performed the CAD analyses. Both raters had more than 10 years of experience in thyroid ultrasound and held specialist certifications recognized by the Colégio Brasileiro de Radiologia e Diagnóstico por Imagem (CBR) (Brazilian College of Radiology and Diagnostic Imaging) and the Associação Médica Brasileira (AMB) (Brazilian Medical Association). Each rater performs approximately 1000 thyroid ultrasound examinations per year. Prior to study initiation, both raters received the same standardized training on the CAD workflow, including image selection and ROI delineation. All analyses were conducted using identical workstation and monitor configurations with the same display settings, under similar workplace conditions.

According to the recommendations of Gerke et al. [11] and the GRRAS guidelines [12], a 90‐day interval between evaluations was adopted to minimize the risk of repeat bias [13], ensuring independence between observations and the validity of reliability estimates. The analyses were conducted on computers in the workplace itself, during routine working hours (7:00 AM–7:00 PM), in accordance with the literature [12]. The evaluating doctors did not receive the patients' history and personal data; that is, there was blinding regarding clinical information, the description of ultrasound findings, and previous ACR TI‐RADS classification, to reduce possible bias. There was no communication between the raters, so they were not aware of each other's analyses; therefore, they were independent. Furthermore, the raters were not aware that their analyses would be compared with those of other raters and with their own after 90 days.

AmCAD‐UT identified the following morphological characteristics of the nodules, according to its system's standard: echogenicity (hyperechoic, isoechoic, hypoechoic, and markedly hypoechoic), echogenic foci (absence, macrocalcification, edge, microcalcification, microcalcification associated with macrocalcification, microcalcification associated with edge), margin (absence, areola, poorly defined, and irregular), shape (oval, round, and irregular), “taller‐than‐wide” (no and yes), texture (homogeneous and heterogeneous), composition (predominantly cystic, predominantly solid, and solid). Purely cystic nodules were not identified by the system. These characteristics will be the variables analyzed, in addition to the ACR TI‐RADS classification and the recommended course of action related to the size of the nodule. Although the system evaluates characteristics that are not considered by ACR TI‐RADS, it establishes the points appropriately, according to ACR TI‐RADS [4, 5, 14].

The information obtained from the analyses conducted by the CAD (at times 1 and 2) was organized in Microsoft Excel spreadsheets for data structuring. The evaluation of intra‐ and inter‐rater reliability was conducted using Cohen's kappa coefficient and the percentage of agreement, with the aim of analyzing the agreement among the evaluating physicians when using CAD in the characterization and classification of thyroid nodule characteristics. The interpretation of the Cohen's kappa coefficient values followed the classification proposed by McHugh [13, 15]. According to this classification, values between 0 and 0.20 indicate the absence of agreement (with only 0%–4% of the data considered reliable). Values between 0.21 and 0.39 are classified as minimal agreement (4%–15% of reliable data), while values from 0.40 to 0.59 correspond to weak agreement (15%–35%). A moderate agreement is attributed to values between 0.60 and 0.79, with 35%–63% reliability. The range between 0.80 and 0.90 represents a strong agreement (64%–81%), and values above 0.90 are considered to indicate almost perfect agreement, with 82% to 100% of the data being deemed reliable. The 95% confidence intervals were obtained using bootstrap resampling. All analyses were conducted in the R programming environment (R Foundation for Statistical Computing, Vienna, Austria), and figures were generated using the ggplot2 package.

## 3 Results

The data regarding the characterization of the 108 patients who presented the 150 evaluated nodules are in Table 1. A total of 150 thyroid nodules were analyzed, obtained through ultrasound examinations conducted at the time of fine‐needle aspiration (FNA) in a private imaging service in the city of São Paulo. The sample included patients of both sexes, aged 18 years or older, with no upper age limit. The selection of cases followed a criterion of consecutive and convenience sampling, with the aim of representing everyday clinical practice. During the study period, there were no exclusions or data losses.

**TABLE 1:** Sample characterization (patients and thyroid nodules).

Variable	*n* = 150
Biological sex	
Female	135 (90%)
Male	15 (10%)
Age (in years)	44 (37; 57)
Size, largest measurement of the nodule (cm)	1.0 (0.80; 1.70)
Location in the thyroid	
Right lobe	74 (49.3%)
Left lobe	67 (44.7%)
Isthmus	9 (6.0%)
Location in the lobe or in the isthmus	
Upper third	19 (12.7%)
Middle third	72 (48.0%)
Lower third	45 (30.0%)
Almost the entire lobe	5 (3.3%)
Right isthmus region	4 (2.7%)
Left isthmus region	3 (2.0%)
Central isthmus region	2 (1.3%)
Composition, according to ACR TI‐RADS	
Cystic—0 points	2 (1.3%)
Mixed (predominantly cystic)—1 point	2 (1.3%)
Mixed (predominantly solid)—1 point	43 (28.7%)
Mixed (solid‐cystic)—1 point	2 (1.3%)
Almost completely cystic—0 points	1 (0.7%)
Almost completely solid—2 points	34 (22.7%)
Solid—2 points	66 (44.0%)
Echogenicity, according to ACR TI‐RADS	
Anechoic—0 points	2 (1.3%)
Hyperechoic—1 point	2 (1.3%)
Isoechoic—1 point	43 (28.7%)
Hypoechoic—2 points	88 (58.7%)
Markedly hypoechoic—3 points	15 (10.0%)
Shape, according to ACR TI‐RADS	
Wider‐than‐tall—0 points	138 (92.0%)
Taller‐than‐wide—3 points	12 (8.0%)
Margin, according to ACR TI‐RADS	
Smooth—0 points	96 (64.0%)
Poorly defined—0 points	5 (3.3%)
Irregular—2 points	43 (28.7%)
Lobulated—2 points	6 (4.0%)
Extrathyroidal extension—3 points	0 (0.0%)
Echogenic foci, according to ACR TI‐RADS	
Comet tail artifact (> 1 mm)—0 points	38 (25.0%)
Absence—0 points	87 (58.0%)
Macrocalcifications (with posterior acoustic shadow)—1 point	7 (4.7%)
Punctate echogenic foci/microcalcifications—3 points	17 (11.0%)
Punctate echogenic foci with macrocalcifications—3 + 1 points	1 (0.7%)
Total points, according to ACR TI‐RADS	4.00 (3.00; 6.00)
Risk category and recommendations, according to ACR TI‐RADS	
TR1 (0 points)—no recommendation	2 (1.3%)
TR1 (1 point)—no recommendation	1 (0.7%)
TR2 (2 points)—no recommendation	20 (13.3%)
TR3 (3 points)—< 1.5 cm: no recommendation	14 (9.3%)
TR3 (3 points)—1.5–2.4 cm: control in 1, 3, and 5 years	9 (6.0%)
TR3 (3 points)—≥ 2.5 cm: FNA	8 (5.3%)
TR4 (4–6 points)—< 1.0 cm: no recommendation	31 (20.7%)
TR4 (4–6 points)—1–1.4 cm: control in 1, 2, 3, and 5 years	16 (10.7%)
TR4 (4–6 points)—≥ 1.5 cm: FNA	20 (13.3%)
TR5 (≥ 7 points)—< 0.5 cm: no recommendation	1 (0.7%)
TR5 (≥ 7 points)—0.5–0.9 cm: annual control for 5 years	12 (8.0%)
TR5 (≥ 7 points)—≥ 1 cm: FNA	16 (10.7%)
ACR TI‐RADS assigned by a human	
1	3 (2.0%)
2	20 (13.0%)
3	31 (21.0%)
4	67 (45.0%)
5	29 (19.0%)
Recommendation, according to ACR TI‐RADS	
FNA	44 (29.0%)
Follow‐up	37 (25.0%)
No recommendation	69 (46.0%)
Bethesda Cytopathology Classification	
I	6 (4.0%)
II	124 (82.7%)
III	12 (8.0%)
IV	3 (2.0%)
V	2 (1.3%)
VI	3 (2.0%)
ACR TI‐RADS assigned by CAD (higher value)	
2	6 (4.0%)
3	18 (12.0%)
4	56 (37.3%)
5	70 (46.7%)
ACR TI‐RADS assigned by CAD (most frequent value, Mode)	
2	6 (4.0%)
3	23 (15.3%)
4	61 (40.7%)
5	60 (40.0%)
Recommendation of FNA by ACR TI‐RADS by the human	
No	106 (70.7%)
Yes	44 (29.3%)
Recommendation for FNA by ACR TI‐RADS by CAD (higher value)	
No	81 (54.0%)
Yes	69 (46.0%)

*Note:* Data are presented as *n* (%) or median (Q1–Q3). No recommendation: without the need for FNA and/or follow‐up.

All the doctors responsible for the ultrasound analyses in the study have solid clinical experience, with over 10 years of practice in diagnoses related to the thyroid gland. These professionals are certified as specialists in Ultrasound by recognized institutions, such as the Brazilian College of Radiology and the Brazilian Medical Association, and have specific training in the application of the ACR TI‐RADS system. Additionally, they are properly trained in the use of the CAD system, especially with the AmCAD‐UT software, developed by AmCad BioMed in Taiwan, ensuring familiarity with the tool and consistency in image interpretation.

**FIGURE 1:**
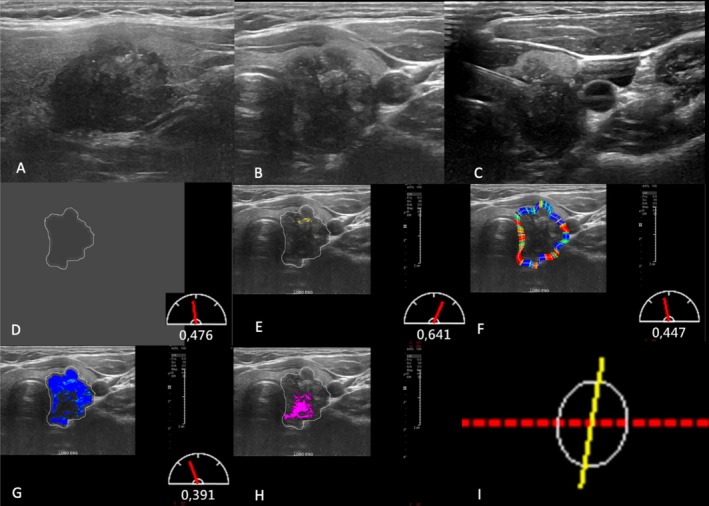
Analysis of a thyroid nodule by the AmCAD‐UT system, after manual delineation. (A) Ultrasonographic image in the longitudinal plane. (B) Ultrasonographic image in the transverse plane. (C) Ultrasonographic image during fine‐needle aspiration of the nodule. (D) Evaluation of echogenicity: Nodule classified as isoechoic (score 0.476). (E) Automatic detection of echogenic foci (in yellow), suggestive of microcalcifications (score 0.641). (F) Margin evaluation, color‐coded according to margin irregularity; margins classified as regular (score 0.447). (G) Texture analysis using color mapping; homogeneous pattern (score 0.391). (H) Identification of anechoic areas (in pink), representing 17.9% of the total area, compatible with a solid‐cystic nodule. (I) Evaluation of the orientation of the axes: Anteroposterior axis greater than the transverse, consistent with a “taller‐than‐wide” morphology (80%). [Color figure can be viewed at wileyonlinelibrary.com]

The analysis of the thyroid nodule (Figure 1) by the AmCAD‐UT platform revealed findings consistent with a high risk of malignancy, although with some discrepancies compared to human evaluation. The figure illustrates the application of the system after the manual delineation of the lesion performed by the operator, a fundamental step that serves as the basis for all subsequent automated analyses. As described in the User's Guide [1], AmCAD‐UT uses this delineation to calculate partial scores related to the main morphological characteristics of the nodule, based on pattern recognition algorithms that express the degree of similarity with malignant findings.

In the illustrated case, the nodule had a maximum diameter of 2.2 cm, being classified by the system as isoechoic (score 0.476), homogeneous (score 0.391), with the presence of echogenic foci interpreted as probable microcalcifications (score 0.641), regular margins (score 0.447), mixed composition (17.9% anechoic), and a taller‐than‐wide shape (80%). Based on these characteristics, AmCAD‐UT assigned 8 points on the ACR TI‐RADS4 scale, classifying the nodule in category 5, with a recommendation for fine‐needle aspiration (FNA). In contrast, the human evaluation described the same nodule as hypoechoic, solid, with irregular margins, evident microcalcifications, and also with a taller‐than‐wide shape, totaling 12 points, a high score within the ACR TI‐RADS 5 category [5].

These differences can be attributed to the inherent subjectivity of human evaluation (especially in the interpretations of echogenicity, composition, and margins), or to the limitations of the CAD algorithm in detecting more subtle morphological patterns. According to the technical guide [1], the system scores are influenced by factors such as intensity calibration, anechoic segmentation, and edge modeling, which can explain part of the observed variations. Despite this, both analyses agreed on the high‐risk classification and the indication for FNA, later confirmed by cytology “suggestive of papillary carcinoma” (Bethesda VI) [16, 17].

**FIGURE 2:**
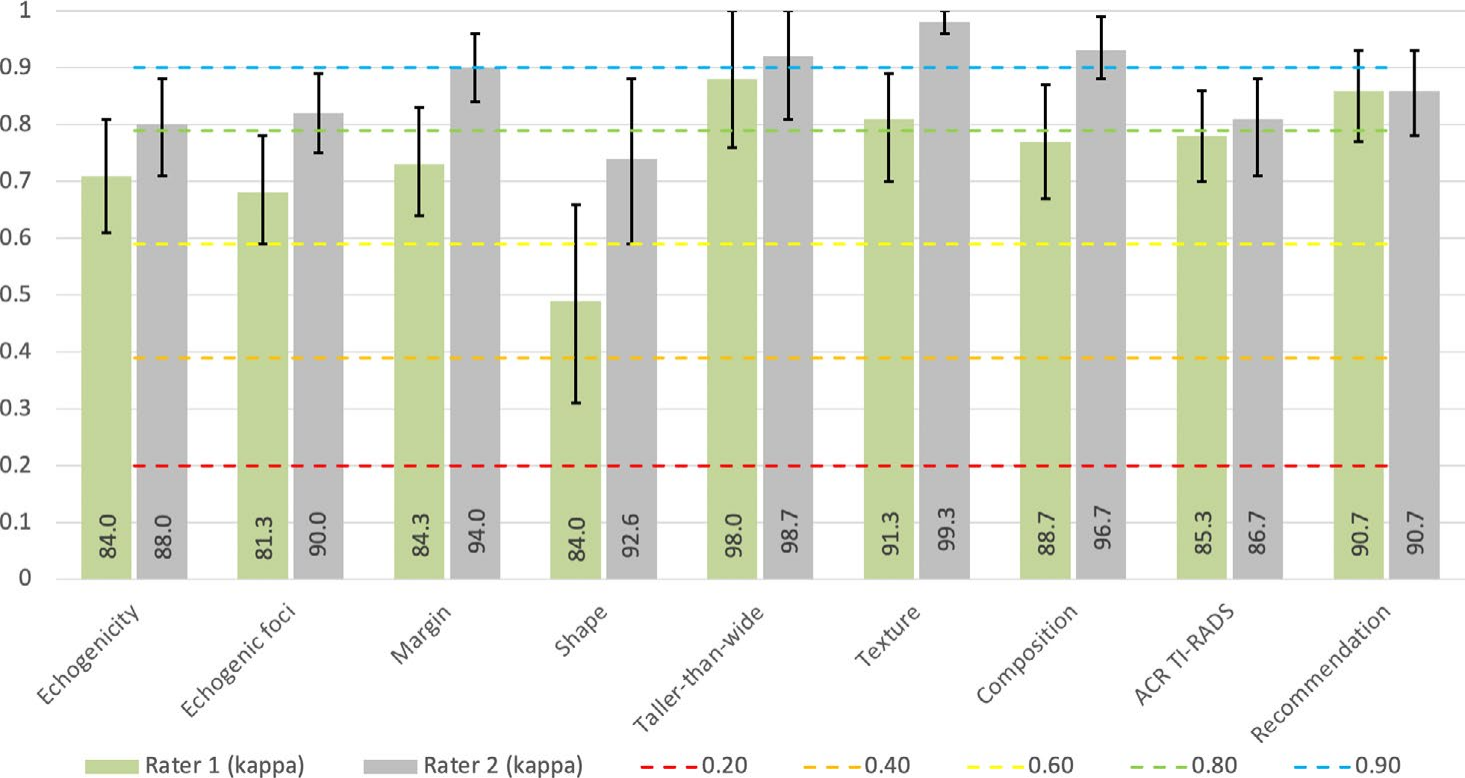
Intra‐rater Cohen's kappa coefficient by variable, obtained using the AmCAD‐UT computer‐aided diagnostic system for the analysis of thyroid nodules. The bars represent the kappa values for raters 1 and 2, with respective error bars indicating the 95% confidence intervals (95% CI). Values displayed at the base of each bar correspond to the percentage of agreement (%C) between the two evaluations conducted by the same observer, with a 90‐day interval. The colored dashed lines represent the limits of the kappa interpretation ranges, according to McHugh [15]: None (≤ 0.20), minimal (0.21–0.39), weak (0.40–0.59), moderate (0.60–0.79), strong (0.80–0.90), and almost perfect (> 0.90). [Color figure can be viewed at wileyonlinelibrary.com]

The intra‐rater reliability of the computer‐aided diagnostic system was evaluated by two doctors, at two different times, with a 90‐day interval between the analyses. The complete results are presented in Table 2 and Figure 2, which summarize the Cohen's kappa coefficients, the 95% confidence intervals, and the agreement percentages obtained for each evaluated variable. The kappa values ranged from 0.49 to 0.88 for rater 1, and from 0.74 to 0.98 for rater 2, indicating agreement that ranges from weak to almost perfect, depending on the variable.

**TABLE 2:** Intra‐rater reliability and agreement in the evaluated variables of thyroid nodules, expressed by the percentage of agreement (%C), Cohen's kappa coefficient, and 95% confidence interval (95% CI), obtained using the AmCAD‐UT computer‐aided diagnosis system—São Paulo, 2025.

Variable	Intra‐rater 1			Intra‐rater 2		
	%C	Kappa	95% CI	%C	Kappa	95% CI
Echogenicity	84.0%	0.71	0.61–0.81	88.0%	0.80	0.71–0.88
Echogenic foci	81.3%	0.68	0.59–0.78	90.0%	0.82	0.75–0.89
Margin	84.3%	0.73	0.64–0.83	94.0%	0.90	0.84–0.96
Shape	84.0%	0.49	0.31–0.66	92.6%	0.74	0.59–0.88
Taller‐than‐wide	98.0%	0.88	0.76–1.00	98.7%	0.92	0.81–1.00
Texture	91.3%	0.81	0.70–0.89	99.3%	0.98	0.96–1.00
Composition	88.7%	0.77	0.67–0.87	96.7%	0.93	0.88–0.99
ACR TI‐RADS	85.3%	0.78	0.70–0.86	86.7%	0.81	0.71–0.88
Recommendation	90.7%	0.86	0.77–0.93	90.7%	0.86	0.78–0.93

*Note:* %C, Percentage of agreement; kappa, Cohen's kappa coefficient; 95% CI (95% confidence interval).

For the echogenicity variable, rater 1 presented a kappa of 0.71 (95% CI: 0.61–0.81), with 84.0% agreement, classified as moderate agreement. Rater 2 obtained a kappa of 0.80 (95% CI: 0.71–0.88) and an agreement of 88.0%, indicating strong agreement.

In the analysis of echogenic foci, rater 1 recorded a kappa of 0.68 (95% CI: 0.59–0.78), with 81.3% agreement, which corresponds to moderate agreement. Rater 2 presented a kappa of 0.82 (95% CI: 0.75–0.89), with 90.0% agreement, classified as strong agreement.

Regarding the margin variable, the kappa was 0.73 (95% CI: 0.64–0.83) for rater 1 and 0.90 (95% CI: 0.84–0.96) for rater 2, with a percentage agreement of 84.3% and 94.0%, respectively. These results correspond to moderate agreement for the first rater and strong agreement for the second.

The variable shape showed a discrepancy between the raters. Rater 1 obtained a kappa of 0.49 (95% CI: 0.31–0.66), indicating weak agreement, with 84.0% agreement. On the other hand, rater 2 obtained a kappa of 0.74 (95% CI: 0.59–0.88), with 92.6% agreement, indicating moderate agreement.

In the evaluation of “taller‐than‐wide,” both raters showed high levels of agreement. Rater 1 obtained a kappa of 0.88 (95% CI: 0.76–1.00), with 98.0% agreement, classified as strong agreement. Rater 2 achieved a kappa of 0.92 (95% CI: 0.81–1.00), with 98.7% agreement, classified as almost perfect agreement.

The texture variable also showed excellent performance. Rater 1 obtained a kappa of 0.81 (95% CI: 0.70–0.89), with 91.3% agreement, indicating strong agreement. Rater 2 obtained a kappa of 0.98 (95% CI: 0.96–1.00), with 99.3% agreement, indicating almost perfect agreement.

For the composition variable, rater 1 presented a kappa of 0.77 (95% CI: 0.67–0.87), with 88.7% agreement, indicating moderate agreement. Rater 2 obtained a kappa of 0.93 (95% CI: 0.88–0.99), with 96.7% agreement, classified as almost perfect agreement.

In the overall classification of ACR TI‐RADS, the kappa was 0.78 (95% CI: 0.70–0.86) for rater 1 and 0.81 (95% CI: 0.71–0.88) for rater 2, with a percentage agreement of 85.3% and 86.7%, respectively. These values correspond to moderate and strong agreement, respectively.

Finally, for the recommendation variable, both raters presented an identical kappa of 0.86 (95% CI: 0.77–0.93), with a percentage agreement of 90.7%, classified as strong agreement according to McHugh [15].

**FIGURE 3:**
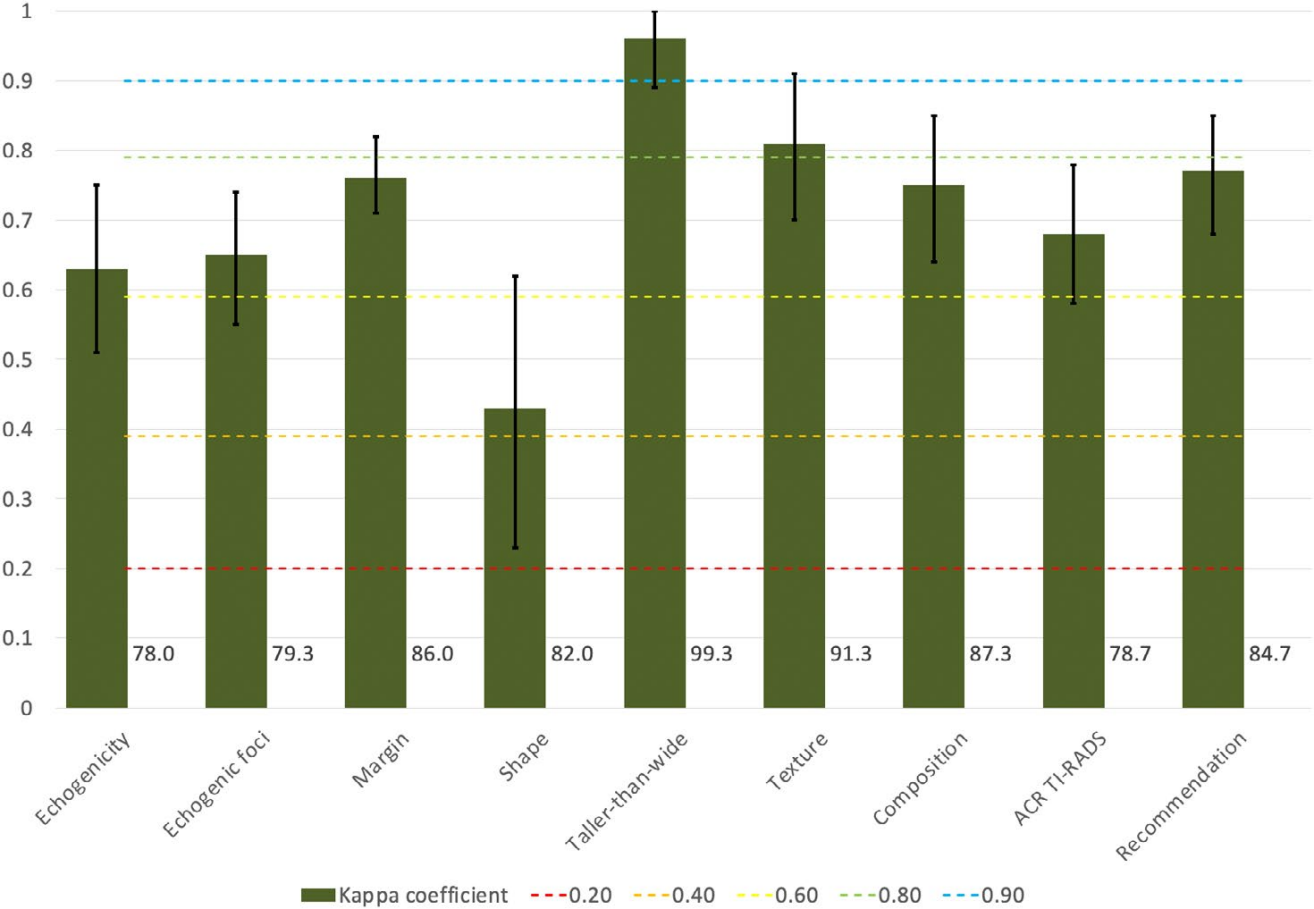
Cohen's kappa inter‐rater coefficient by variable, at time 1, obtained using the AmCAD‐UT computer‐aided diagnostic system for the analysis of thyroid nodules. The bars represent the kappa values obtained between the raters, with respective error bars indicating the 95% confidence intervals (95% CI). Values displayed at the base of each bar correspond to the percentage of agreement (%C) between the two raters in the analysis of each variable. The colored dashed lines represent the limits of the kappa interpretation ranges, according to McHugh [15]: None (≤ 0.20), minimal (0.21–0.39), weak (0.40–0.59), moderate (0.60–0.79), strong (0.80–0.90), and almost perfect (> 0.90). [Color figure can be viewed at wileyonlinelibrary.com]

Inter‐rater reliability was specifically analyzed at time 1, based on the comparison between the classifications assigned by two different doctors using the computer‐aided diagnostic system, applied to the same images of thyroid nodules. The results, organized in Table 3 and Figure 3, include Cohen's kappa coefficients, 95% confidence intervals, and the percentages of agreement for each evaluated variable, allowing verification of the degree of reproducibility of the system among different observers.

**TABLE 3:** Inter‐rater reliability and agreement in the evaluated variables of thyroid nodules, expressed by the percentage of agreement (%C), Cohen's kappa coefficient, and 95% confidence interval (95% CI), obtained using the AmCAD‐UT computer‐aided diagnostic system—São Paulo, 2025.

Variable	Inter‐rater		
	%C	Kappa	CI
Echogenicity	78.0%	0.63	0.51–0.75
Echogenic foci	79.3%	0.65	0.55–0.74
Margin	86.0%	0.76	0.71–0.82
Shape	82.0%	0.43	0.23–0.62
Taller‐than‐wide	99.3%	0.96	0.89–1.00
Texture	91.3%	0.81	0.70–0.91
Composition	87.3%	0.75	0.64–0.85
ACR TI‐RADS	78.7%	0.68	0.58–0.78
Recommendation	84.7%	0.77	0.68–0.85

*Note:* %C, Percentage of agreement; kappa, Cohen's kappa coefficient; 95% CI, 95% confidence interval.

For the echogenicity variable, a percentage agreement of 78.0% was observed, with a kappa of 0.63 (95% CI: 0.51–0.75), indicating moderate agreement among the raters. Regarding the echogenic foci, the concordance was 79.3%, with a kappa of 0.65 (95% CI: 0.55–0.74), also classified as moderate.

In the evaluation of the margin variable, the assessors showed 86.0% agreement and a kappa of 0.76 (95% CI: 0.71–0.82), which characterizes a higher moderate agreement, close to the upper limit of the category. The variable shape presented the lowest kappa value among all the analyzed variables, with 82.0% agreement and a kappa of 0.43 (95% CI: 0.23–0.62), being classified as weak agreement.

The variable “taller‐than‐wide” achieved the best inter‐rater indices, with an agreement of 99.3% and a kappa of 0.96 (95% CI: 0.89–1.00), representing almost perfect agreement. Similarly, the texture variable showed a kappa of 0.81 (95% CI: 0.70–0.91), with an agreement of 91.3%, classified as strong agreement.

For the composition variable, an agreement of 87.3% and a kappa of 0.75 (95% CI: 0.64–0.85) were observed, a result indicating moderate agreement. In the ACR TI‐RADS variable, the raters presented a kappa of 0.68 (95% CI: 0.58–0.78), with 78.7% agreement, also classified as moderate.

Finally, in the recommendation variable, the kappa was 0.77 (95% CI: 0.68–0.85), with a percentage agreement of 84.7%, which represents moderate agreement in the upper range of the classification.

## 4 Discussion

The evaluation of the reliability of CAD systems is fundamental to validate their use as a complementary tool in clinical practice. In a scenario like thyroid ultrasound, where the analysis is notoriously operator‐dependent, the possibility of standardization through automated algorithms represents a significant advancement [18]. However, the adoption of any technology in routine care requires evidence that its results are consistent when used by different professionals and at different times. This study was conducted with the aim of systematically measuring the intra‐ and inter‐rater reproducibility of a CAD system applied to the evaluation of thyroid nodules, providing concrete data on its performance in the face of the inherent human variability in medical practice.

Interobserver variability among human raters is a well‐recognized challenge in thyroid ultrasound. In most studies, agreement is quantified using kappa statistics, and the overall picture is consistent: reliability is typically moderate at best, with substantial heterogeneity across individual sonographic features and across risk‐stratification systems. When readers apply widely used classification systems (such as ACR TI‐RADS, ATA, EU‐TIRADS, or K‐TIRADS) pooled interrater agreement for the final category assignment is generally moderate, with kappa values commonly reported around 0.5, meaning that different clinicians often reach only partial concordance when classifying the same nodule. Importantly, agreement is not uniform across features. Some characteristics tend to show higher reproducibility (e.g., nodule composition and certain calcification patterns, particularly when recorded as present/absent), whereas others are consistently less reliable, especially margins, echogenic foci, echogenicity, and shape. Margin assessment is a notable example, frequently demonstrating low agreement in published series (kappa as low as approximately 0.14–0.34), likely reflecting the need for subjective interpretation of contour irregularity. In contrast, composition and the presence/absence of calcification patterns may reach substantial agreement in some cohorts (approximately up to 0.6–0.78). Variability between observers is influenced by reader‐related and system‐related factors, including professional experience, the presence of structured training, and the clarity and operationalization of feature definitions; standardized lexicons and consensus‐oriented training can improve reproducibility, and comparative studies suggest that differences in experience may change how strictly explicit criteria are applied. As expected, intraobserver agreement (the same reader reassessing images at different time points) is usually higher than interobserver agreement. Taken together, these findings highlight the clinical importance of reliability, because variability in feature interpretation can propagate to the final risk category and directly affect downstream management decisions, including biopsy referral [19, 20, 21, 22, 23, 24].

The intra‐rater analysis of the first doctor revealed significant variation in the levels of agreement among the different morphological variables assessed, with kappa coefficients ranging from 0.49 to 0.88. The variables “taller‐than‐wide” (kappa = 0.88; 95% CI: 0.76–1.00), “recommendation” (0.86; 95% CI: 0.77–0.93), and “texture” (0.81; 95% CI: 0.70–0.89) showed strong agreement, with agreement percentages exceeding 90%, reflecting greater consistency of the system for these characteristics. The variables “ACR TI‐RADS” (0.78; 95% CI: 0.70–0.86), “composition” (0.77; 95% CI: 0.67–0.87), “margin” (0.73; 95% CI: 0.64–0.83), “echogenicity” (0.71; 95% CI: 0.61–0.81), and “echogenic foci” (0.68; 95% CI: 0.59–0.78) were classified as having moderate agreement, with agreement percentages between 81.3% and 88.7%. Finally, the variable “shape” showed the lowest kappa value, with 0.49 (95% CI: 0.31–0.66), being interpreted as weak agreement, despite a high percentage of agreement of 84.0%.

This result highlights a critical point in reliability analysis: high percentages of agreement do not always reflect high reproducibility, especially when there is an imbalance in the distribution of categories. This was observed, for example, in the variable “shape,” which presented a kappa of 0.49 (95% CI: 0.31–0.66) indicating weak agreement, despite a percentage of agreement of 84.0%. This apparent contradiction occurs because simple percentage agreement does not consider the agreement expected by chance, potentially overestimating the real consistency of the method. The kappa coefficient statistically adjusts this value, providing a more rigorous estimate of the stability between measurements. Thus, even when the raters agree in most cases, if this agreement is concentrated in very prevalent categories or in classifications that are not very sensitive to discrete variations, the kappa tends to remain lower. This distinction reinforces the importance of using multiple metrics to interpret the reproducibility of automated systems, especially in diagnostic contexts that require clinical decisions based on subtle morphological nuances.

The intra‐rater analysis of the second doctor revealed consistently high performance of the computer‐aided diagnostic system, with kappa coefficients ranging from 0.74 to 0.98. The variables “texture” (kappa = 0.98; 95% CI: 0.96–1.00), “composition” (0.93; 95% CI: 0.88–0.99), and “taller‐than‐wide” (0.92; 95% CI: 0.81–1.00) showed almost perfect agreement, with agreement percentages of 99.3%, 96.7%, and 98.7%, respectively. The variables “margin” (0.90; 95% CI: 0.84–0.96), “recommendation” (0.86; 95% CI: 0.78–0.93), “echogenic foci” (0.82; 95% CI: 0.75–0.89), and “ACR TI‐RADS” (0.81; 95% CI: 0.71–0.88) were classified as having strong agreement, all with a percentage agreement equal to or greater than 86.7%.

The variable “shape” presented the lowest kappa for this rater, with 0.74 (95% CI: 0.59–0.88), being classified as having moderate agreement, despite the high observed percentage agreement (92.6%). This pattern reinforces the previously observed finding with rater 1, suggesting that the asymmetric distribution of categories may negatively impact the interpretation of kappa. Overall, the indices obtained by the second rater were higher, which may reflect greater familiarity with the system or a more stable judgment pattern. Even so, the data reaffirm that the performance of CAD tends to be more consistent in variables of a dichotomous nature and with less subjectivity, reinforcing its role as a complementary tool to clinical analysis, and not as a substitute for medical interpretation.

When comparing the intra‐rater results of the two doctors, it is observed that the second rater generally presented higher kappa coefficients and narrower confidence intervals, suggesting greater stability and reproducibility in the analyses conducted with the CAD system. While the first rater obtained kappa values ranging from 0.49 to 0.88, the second presented values between 0.74 and 0.98. The greatest discrepancies occurred in the variable “composition” (kappa = 0.77 for rater 1 and 0.93 for rater 2), “texture” (0.81 vs. 0.98), and “shape” (0.49 vs. 0.74). On the other hand, some variables showed high and stable performance in both raters, such as “taller‐than‐wide” and “recommendation,” suggesting that the system tends to exhibit greater reliability in morphologically more objective or dichotomous characteristics.

Although both raters had similar clinical experience and standardized training in using the system, subtle differences in image selection and manual ROI delineation may have influenced the results. Because the CAD workflow depends on operator‐performed steps prior to automated processing, small variations in contouring or image choice can affect feature extraction and, consequently, agreement estimates. These findings highlight the importance of standardizing critical operational steps, particularly for variables that involve greater visual interpretation, in order to minimize user‐dependent variability and improve reproducibility. Thus, while both raters achieved globally satisfactory results, differences in kappa magnitude indicate that reproducibility depends not only on the technology itself but also on how it is operationalized in clinical practice. The inter‐rater analysis conducted at time 1 demonstrated that the reliability between the two raters varied significantly among the analyzed variables, with kappa coefficients ranging from 0.43 to 0.96. The variable “taller‐than‐wide” showed the best performance, with kappa = 0.96 (95% CI: 0.89–1.00), classified as almost perfect agreement, and a percentage agreement of 99.3%. The variable “texture” also achieved strong agreement, with kappa = 0.81 (95% CI: 0.70–0.91) and 91.3% agreement.

The variables “recommendation” (kappa = 0.77; 95% CI: 0.68–0.85), “margin” (0.76; 95% CI: 0.71–0.82), “composition” (0.75; 95% CI: 0.64–0.85), “ACR TI‐RADS” (0.68; 95% CI: 0.58–0.78), “echogenic foci” (0.65; 95% CI: 0.55–0.74), and “echogenicity” (0.63; 95% CI: 0.51–0.75) showed moderate agreement, with agreement percentages between 78.0% and 87.3%. The variable “shape” obtained the lowest kappa, with 0.43 (95% CI: 0.23–0.62), being classified as having weak agreement, despite a concordance percentage of 82.0%.

This pattern reinforces previously observed findings in the intra‐rater analysis, indicating that variables with greater subjectivity or multiple possible categories tend to exhibit lower consistency among observers, even with the use of an automated system. As previously discussed, the discrepancy between high percentage agreement and lower kappa values in the “shape” variable reflects the influence of category imbalance and reinforces the importance of interpreting both metrics jointly. Even with experienced and equally trained raters, inter‐rater variability remains relevant, and it shows that the performance of CAD can be influenced by subjective decisions, such as the choice of the representative image or the manual delineation of the nodule. These findings reinforce the need for rigorous standardization in the operational stages of using the tool, especially in scenarios with multiple operators sharing diagnostic decisions.

An additional factor that may have influenced the lower reproducibility observed for “shape” relates to the classification framework adopted by the CAD system. Unlike ACR TI‐RADS, which operationalizes shape primarily through the dichotomous “taller‐than‐wide” descriptor, AmCAD‐UT categorizes shape into oval, round, and irregular patterns as a separate variable. This finer morphological granularity may increase interpretative variability and partially explain the lower kappa values observed for this feature.

Overall, the findings demonstrate that the computer‐aided diagnostic system achieved predominantly moderate to strong reproducibility, with several variables reaching strong or almost perfect agreement. The intra‐rater reliability was mostly classified as moderate to strong, with superior performance in dichotomous variables such as “taller‐than‐wide.” Inter‐rater reliability showed greater variability across features, ranging from weak agreement for “shape” to almost perfect agreement for “taller‐than‐wide,” with most variables demonstrating moderate to strong reproducibility. These findings suggest that, although CAD contributes to evaluation standardization, its performance can still be influenced by operational and human factors. The repetition of low agreement patterns in variables such as “shape,” even with the use of an automated tool, indicates that the standardization promoted by CAD has limitations, especially when applied to attributes with greater interpretative subjectivity. This suggests that there are areas that can be improved, such as the evaluation of nodular morphology, which showed greater variability among professionals.

Future developments in segmentation strategies and morphology‐sensitive modeling may enhance consistency in features that demonstrated lower reproducibility, particularly shape. These findings, although promising, reinforce that the use of CAD should be understood as a complementary support to clinical reasoning, and not as a substitute for qualified medical analysis. The responsible adoption of technology should be accompanied by well‐defined protocols, adequate training of operators, and recognition of circumstances that may interfere with the reproducibility of results.

This study presents some limitations that must be considered in the interpretation of the results. First, this is a single‐center study, conducted in a single imaging service, with a sample composed of 150 consecutively obtained thyroid nodules. Although this number was sufficient to estimate the reliability coefficients with reasonable accuracy, the lack of representativeness of different clinical contexts, geographical regions, and population profiles limits the generalizability of the findings. The conduct of multicentric studies, with institutional and demographic diversity, could help validate the results in broader and more heterogeneous settings, enhancing the generalization and clinical application potential of CAD. Referral for FNA was determined by the patients' treating physicians in routine care, and the criteria used for referral (including the use of ACR TI‐RADS thresholds) were not uniformly documented.

Additional limitations relate to the operational dependency inherent to the CAD workflow. In this study, analyses were performed on still B‐mode images and required manual delineation of the nodule contour (ROI) by the operator, as well as selection of the most representative image for upload. These steps may introduce user‐dependent variability and may partially explain differences observed between raters, even under standardized training and identical workstation settings. Moreover, ultrasound is inherently dynamic, and real‐time scanning—including examinations performed by surgeons in clinical settings—may provide additional information that is not fully captured by still images. Because our CAD assessments were restricted to static images obtained immediately before FNA, generalizability to workflows that rely on cine‐loops or real‐time scanning may be limited. Although most commercially available thyroid CAD tools have primarily been developed and deployed using still images, research on cine‐loop and video‐based AI approaches is emerging; however, their integration into standardized clinical workflows and guidelines remains limited and requires further prospective validation [25, 26, 27, 28, 29].

Furthermore, despite the raters having similar clinical experience and standardized training in using the system, the impact of individual operational factors, such as the accuracy in manually delineating the nodules or the interaction with the software interface, cannot be disregarded. Such aspects, although difficult to measure directly, may have influenced the intra‐ and inter‐rater results observed. Furthermore, the evaluation was based on static images, previously selected, which do not fully reflect the dynamics of real clinical practice, where the operator directly obtains and chooses the images.

Finally, this study focused exclusively on analyzing the reliability of the CAD system for the morphological characterization of nodules. Therefore, the results should be interpreted within the proposed methodological scope, recognizing both the advances and the limitations of the approach used.

So far, most of the available publications on computer‐aided diagnostic systems for the evaluation of thyroid nodules focus on diagnostic performance metrics (such as sensitivity, specificity, and accuracy) or on inter‐rater reliability studies, with an emphasis on the comparison between CAD and human observers. Although several studies have examined inter‐rater agreement or diagnostic performance of CAD systems, intra‐rater reliability assessed through repeated temporal evaluations remains insufficiently explored. This methodological gap supports the originality of the present study by proposing a structured approach to measure the intra‐rater reproducibility of CAD, applying two independent evaluations with a temporal interval and robust statistical analysis. The inclusion of this dimension allows for an expanded understanding of the system's stability in real clinical use situations, contributing in an original way to the field of technological assessment in thyroid ultrasound.

Similar evaluations of CAD reproducibility for thyroid nodule assessment have been reported in international cohorts across different healthcare settings, enabling contextual comparison of agreement estimates across platforms and study designs. Several international studies have evaluated the inter‐rater reliability of CAD systems, such as AmCAD‐UT (AmCad BioMed, Taiwan) and S‐Detect for Thyroid (Samsung Medison, Korea), applied to the risk stratification of thyroid nodules, with results ranging from minimal to almost perfect agreement, depending on the variable analyzed and the context of use.

To facilitate comparison across platforms and study designs, Table 4 summarizes the main international studies that evaluated inter‐ and/or intra‐rater reliability of CAD systems for thyroid nodule assessment, including the present study. This approach allows the reader to visualize feature‐specific agreement patterns without repeating all kappa values in the narrative text.

**TABLE 4:** Summary of international studies evaluating inter‐ and/or intra‐rater reliability of CAD systems for thyroid nodule assessment (chronological order).

Study (Year)	CAD platform	Study design	Sample size	Kappa range	Variables with higher agreement	Variables with lower agreement	Key findings
Choi et al. (2017)	S‐Detect	P	200	0.24–0.74	Shape, orientation, composition	Margins	Moderate–strong agreement for structural features; lower agreement for margins
Gitto et al. (2019)	S‐Detect	R	117	0.03–0.69	Orientation	Margins, echogenicity	Marked variability across morphological features
Jeong et al. (2019)	CAD system	P	209	0.65–0.79	Spongiform, echogenicity	Margins	Agreement influenced by reader experience
Kim et al. (2019)—S‐Detect 1	S‐Detect 1	R	218	0.36–0.80	Composition	Margins; calcification	Higher agreement for composition; lower reproducibility for margins
Kim et al. (2019)—S‐Detect 2	S‐Detect 2	R	218	0.34–0.65	Composition	Calcification	Persistent variability in calcification assessment
Lu et al. (2019)	AmCAD‐UT	R	190	0.29–0.88	Nodules < 1 cm	1–2 cm nodules	Agreement varied according to nodule size
Reverter et al. (2019)	AmCAD‐UT	R	145	0.33–0.54	Overall TI‐RADS	Benign nodules	Weak–moderate agreement; lower consistency in benign nodules
Li et al. (2020)	AmCAD‐UT	P	172	0.33–0.78	Taller‐than‐wide	Microcalcifications; heterogeneous echotexture	Lower reproducibility in morphology‐dependent features
Present Study (2024)	AmCAD‐UT v2.2	P	150	0.43–0.98	Taller‐than‐wide, texture, composition	Shape	Moderate–almost perfect intra‐rater; moderate–strong inter‐rater reproducibility

Abbreviations: P = prospective; R = retrospective.

Choi et al., using S‐Detect, observed moderate to strong agreement between the CAD system and an experienced radiologist for shape, orientation, and composition, but reported minimal agreement for margins [30]. Gitto et al., also evaluating S‐Detect, described moderate agreement for orientation, but minimal or no agreement for echogenicity and margins [31]. Jeong et al. reported overall moderate agreement among raters with different levels of experience using CAD, with better performance for characteristics such as spongiform pattern, echogenicity, and orientation [32].

In the study by Kim et al., interobserver agreement between radiologists and S‐Detect demonstrated variable performance across features. For both S‐Detect versions, composition showed the highest agreement, whereas margins and calcification‐related features exhibited lower reproducibility, reinforcing the variability observed in morphology‐dependent descriptors (Table 4) [33].

Among studies specifically evaluating AmCAD‐UT, agreement estimates have also varied according to feature and clinical context (Table 4). Lu et al. reported overall moderate agreement, with better reproducibility in smaller nodules, whereas larger nodules showed a decline in consistency [34]. Li et al. demonstrated stronger agreement for orientation and hypoechogenicity, but lower reproducibility for margins, microcalcifications, and heterogeneous texture, consistent with the greater subjectivity of these features [35]. Reverter et al. observed overall weak agreement, particularly in benign nodules, suggesting that CAD performance may be more limited in low‐suspicion scenarios [36].

When contextualized within the existing literature (Table 4), the present study demonstrates agreement estimates that are comparable to, and in some domains higher than, those reported for both AmCAD‐UT and S‐Detect platforms. In particular, reproducibility was consistently stronger for more objective features such as orientation (“taller‐than‐wide”) and texture, whereas lower agreement for shape aligns with the broader literature, in which morphology‐dependent descriptors remain among the most challenging features to standardize. These findings suggest that, while CAD systems contribute to reducing variability, certain sonographic characteristics continue to depend on interpretative nuances that are not fully mitigated by automated analysis.

Overall, our inter‐rater kappa values ranged from 0.43 to 0.96 across variables, while intra‐rater kappa ranged from 0.49 to 0.88 for rater 1 and 0.74 to 0.98 for rater 2. These estimates fall within, and in some domains appear higher than, the range reported in prior international studies, particularly for more objective features (e.g., orientation/taller‐than‐wide and texture). Conversely, lower agreement for shape in our analyses is consistent with the broader literature, in which morphology‐related descriptors tend to be among the least reproducible features, even with CAD assistance.

## 5 Conclusions

The present study demonstrated that the computer‐aided diagnostic system achieved predominantly moderate to almost perfect intra‐rater reliability and moderate to strong inter‐rater agreement across most morphological variables of thyroid nodules. Inter‐rater reproducibility varied across features, ranging from weak agreement for “shape” to almost perfect agreement for “taller‐than‐wide,” while the majority of variables showed consistent moderate to strong performance. These findings support the system's role as a reliable adjunct for the standardization of thyroid ultrasound evaluation. Nevertheless, its performance remains partially influenced by operator‐dependent factors, underscoring the importance of standardized protocols, adequate training, and further multicenter validation.

## Conflicts of Interest

The authors declare no conflicts of interest.

## Data Availability

The data that support the findings of this study are available from the corresponding author upon reasonable request.
